# Immediate release oral nifedipine for severe hypertension in pregnancy—Time for re‐prioritization as a management option

**DOI:** 10.1002/pmf2.70068

**Published:** 2025-07-04

**Authors:** Minhazur Sarker, Timothy Wen, E. Nicole Teal, Ukachi N. Emeruwa

**Affiliations:** ^1^ Division of Maternal‐Fetal Medicine Department of Obstetrics Gynecology and Reproductive Science University of California San Diego San Diego California USA; ^2^ Division of Biomedical Informatics Department of Medicine University of California San Diego San Diego California USA

## 1 INTRODUCTION

Hypertensive disorders of pregnancy affect up to 8% of pregnancies globally and account for a significant number of maternal deaths worldwide [1]. The incidence of preeclampsia has also risen by 25% over the past two decades, resulting in considerably more pregnant patients presenting with severe hypertension in pregnancy [1]. While preeclampsia and the associated severe hypertension are often transient, delayed or suboptimal management may result in adverse outcomes including placental abruption, stroke, myocardial infarction, aortic dissection, vascular injury, or renal injury [1]. Thus, blood pressure (BP) control remains a key component of the management of preeclampsia.

## 2 CURRENT MANAGEMENT PARADIGM

The American College of Obstetricians and Gynecologists (ACOG) Committee Opinion #692—“Emergent Therapy for Acute‐Onset, Severe Hypertension During Pregnancy and the Postpartum Period” highlights the clinical evidence and management algorithms followed by providers in the United States [1]. Severe hypertension, defined as systolic BP ≥ 160 mmHg or diastolic BP ≥ 110 mmHg, should ideally be managed within 30–60 min. Current management options for acute severe hypertension include intravenous (IV) labetalol, IV hydralazine, and oral immediate release (IR) nifedipine. Despite a Cochrane systematic review revealing equal efficacy and safety of all three agents, the ACOG committee opinion states that IV labetalol and hydralazine should be considered first‐line therapy while IR nifedipine may be considered when IV access is not available [1, 2]. A summary of management options for acute severe hypertension from other professional societies is summarized in Table 1.

**TABLE 1:** Summary of first‐line anti‐hypertensive options for management of acute severe hypertension in pregnancy.

American College of Obstetricians and Gynecologists (ACOG) [1]	National Institute for Health and Care Excellence (NICE/RCOG) [3]	Society of Obstetric Medicine of Australia and New Zealand (SOMANZ) [4]	Society of Obstetricians and Gynaecologists of Canada (SOGC) [5]	World Health Organization (WHO) [6]
IV Labetalol	IV Labetalol	IV Labetalol	IV Labetalol	IV Labetalol
IV Hydralazine	IV Hydralazine	IV Hydralazine	IV Hydralazine	IV Hydralazine
IR Nifedipine	IR Nifedipine	IR Nifedipine	IR Nifedipine	IR Nifedipine
		Oral Methyldopa	Oral Methyldopa	Oral Methyldopa
		Oral Labetalol	Oral Labetalol	
		IV Diazoxide		

A recent study evaluated the temporal trends in antihypertensive medication use during delivery hospitalizations for preeclampsia and found that from 2006 to 2015, the proportion of patients receiving any anti‐hypertensive medications rose from 37.8% to 49.4% [7]. Moreover, from 2006 to 2015, receipt of oral labetalol increased from 20.3% to 31.4%, IV labetalol from 13.3% to 21.4%, IV hydralazine from 12.8% to 16.9%, oral nifedipine (including IR and extended release formulation) from 15.0% to 18.2%, and the use of more than one medication from 16.5% to 25.8% [7]. In other words, during the same study period, there was a 54.7% increase in the use of oral labetalol, 60.9% of IV labetalol, 21.3% of IV hydralazine, and 32.0% of oral nifedipine. Thus, this large national data analysis suggests preferential utilization of labetalol over hydralazine and nifedipine for the acute management of hypertensive disorders of pregnancy.

## 3 A CASE FOR CHANGE

Although the ACOG committee opinion does highlight that some studies suggest IR nifedipine lowers BP more efficiently than other options, the guideline has not been updated with newer literature [1]. More recently, there have been multiple randomized controlled trials (RCTs) and systematic reviews that have better characterized the clinical utility of IR nifedipine [8, 9, 10, 11, 12]. In this article, we will review the recent literature on IR nifedipine in managing severe hypertension in pregnancy. We believe that these new data warrant a re‐evaluation of the above ACOG committee opinion to more clearly indicate equal efficacy of IR nifedipine regardless of the presence of IV access.

## 4 RECENT LITERATURE FOR IR NIFEDIPINE VERSUS IV LABETALOL

### 4.1 Shekhar et al. [8]

In 2016, this systematic review and meta‐analysis aimed to compare the safety and efficacy of IR nifedipine compared to IV labetalol for the treatment of severe hypertension during pregnancy. It included all RCTs comparing IR nifedipine to IV labetalol and created a pooled analysis of seven different trials (four of which were from low‐resource countries), resulting in a cohort of 363 patients. IR nifedipine compared to IV labetalol was associated with lower risk of persistent hypertension defined as either the need for non‐allocated antihypertensive drug or as failure to lower the BP to safe levels with the allocated treatment (relative risk (RR) 0.42, 95% CI 0.18–0.96) or maternal side effects (RR 0.57, 95% CI 0.35–0.94) with the conclusion that IR nifedipine was at least as efficacious and safe as IV labetalol and may be more accessible in low‐resource settings.

### 4.2 Shi et al. [9]

In 2016, another RCT was performed at the Cangzhou Central Hospital in China comparing IR nifedipine versus IV labetalol for the treatment of severe hypertension with severe preeclampsia. The trial enrolled 147 patients and allocated 1:1 to receive either IR nifedipine or IV labetalol to achieve the target BP (< 150/100 mmHg). The primary outcome was time to achieve the target BP and secondary outcomes included time interval before next hypertensive crisis by BP (≥160/110 mmHg), number of doses to achieve target BP, and adverse events. In the trial, the time to achieve the target BP was not statistically different (35 min for IR nifedipine vs. 42 min for IV labetalol, *p* = 0.37). Additionally, no statistical difference was found in the time interval to the next hypertensive crisis, number of drug dosages to achieve target BP, or adverse events. Thus, the authors concluded that IR nifedipine was equally as effective as IV labetalol in the management of acute hypertension in pregnancy.

### 4.3 Li et al. [11]

In 2023, a second systematic review and meta‐analysis was performed, incorporating contemporary literature focusing on the time to achieve each respective study's target BP (the majority utilized ≤ 150/100 mmHg). Secondary outcomes included number of doses required to achieve target BP and adverse maternal or neonatal effects. This analysis included 12 RCTs creating a pooled cohort of 1151 participants. Of note, among the twelve RCTs, nine were conducted in India while one each was in China, the United States, and Malaysia. Participants receiving IR nifedipine reached the target BP more expeditiously (mean difference 7.64 min, 95% CI 4.08–11.20, *p* < 0.01) than those receiving IV labetalol. Among the 10 RCTs commenting on the number of doses, significantly fewer doses of IR nifedipine were required to achieve the target blood pressure (mean difference 0.62 doses, 95% CI 0.36–0.88, *p* < 0.01). No differences were noted in maternal or neonatal complications. Thus, they concluded that IR nifedipine worked more quickly and required fewer doses without adverse effects.

## 5 RECENT LITERATURE FOR ALL THREE ACUTE ANTI‐HYPERTENSIVE MEDICATIONS

### 5.1 Donel et al. [10]

While prior studies have focused on IR nifedipine versus IV labetalol, this RCT included a third arm using IV hydralazine. Sixty patients at Universitas Riau in Indonesia were randomized 1:1:1 to receive anti‐hypertensive medications; BP and mean arterial pressure (MAP) were measured for 5 h post‐intervention. The effectiveness of each medication was assessed by the ability to reduce the MAP by 20% within 20 min after a single dose and after the three‐dose series. After a single dose, 20% MAP reduction was achieved with IR nifedipine by 57.5% of participants, with IV labetalol by 42.1% of participants, and with IV hydralazine by 40.9% of participants (*p* = 0.048). After the third dose, 5% of IV hydralazine participants, 15% of oral nifedipine participants, and 35% of IV labetalol participants failed to reach the MAP reduction target (*p* = 0.044). Thus, the authors concluded that IR nifedipine was more rapid in its effect, IV hydralazine was the most effective overall, and IV labetalol was least effective and had the highest chance of failure, although the study had a small sample size.

### 5.2 Bhat et al. [12]

Given the lack of consensus on the relative efficacy of anti‐hypertensives in pregnancy, this systematic review and meta‐analysis aimed to compare treatment success between all medications available including IR nifedipine, sublingual nifedipine, IV hydralazine, IV labetalol, and other medications (such as IV urapidil, oral methyl‐dopa, oral prazosin, etc.) that have since fallen out of favor. In total, 74 RCTs were included with 8324 pooled participants receiving treatment for severe hypertension as defined by each study (inclusive of all hypertensive disorders of pregnancy and chronic hypertension). The primary outcome was treatment success defined as achieving the target BP designated by each study. The study showed that IR nifedipine and sublingual nifedipine were most likely to be effective at achieving the target BP. However, after sub‐grouping for the presence of preeclampsia, IR nifedipine was the most effective. Thus, they concluded that IR nifedipine was the most efficacious.

## 6 MATERNAL RISKS WITH IR NIFEDIPINE

While IR nifedipine is typically safe, the non‐specific vasodilatory effect as a dihydropyridine calcium channel blocker may cause rare but serious adverse effects. Case reports have connected IR nifedipine to myocardial infarction, heart failure, and coronary steal syndrome [13, 14, 15, 16]. Although the greatest susceptibility is seen in patients with cardiovascular disease, many patients with severe preeclampsia also have significant cardiovascular risk factors, warranting individualized risk‐benefit consideration and caution with use. These rare adverse events are unlikely to be captured by smaller RCTs or even the meta‐analyses conducted.

## 7 FUTURE DIRECTION

As outlined above, there is a growing body of evidence indicating IR nifedipine is at least as efficacious as IV labetalol and IV hydralazine both in time to achieve target BP and number of doses required. Based on this evidence, IR nifedipine should be considered an equally efficacious first‐line therapy for acute hypertension in pregnancy.

Furthermore, we believe there is adequate evidence to support a change to preferential utilization of IR nifedipine for severe hypertension in pregnancy except in the case of imminent operative cesarean delivery or when oral intake is otherwise contraindicated. Moreover, from a management perspective, IR nifedipine permits an easy dosing schedule, can be administered orally, is readily available and cost‐efficient, and therefore, is well suited for use in resource‐poor settings.

## CONFLICT OF INTEREST STATEMENT

T.W. serves on the medical advisory board for Delfina Care, Inc. and retains equity and a stipend as compensation. The work presented here is not related to his work with Delfina Care, Inc. All other authors declare no conflicts of interest.

## DISCLOSURES

This work has never been presented. No component of this original manuscript has previously been published elsewhere and is not under consideration for publication in any journal aside from the *Pregnancy* Journal.

## Data Availability

No original data were utilized for the formulation of this clinical opinion.
